# Knockout of IL-6 mitigates cold water-immersion restraint stress-induced intestinal epithelial injury and apoptosis

**DOI:** 10.3389/fimmu.2022.936689

**Published:** 2022-11-25

**Authors:** Yuan Zhang, Chujun Duan, Shuwen Wu, Jingchang Ma, Yongming Liu, Wenpeng Li, Tingting Wang, Lu Yang, Kun Cheng, Ran Zhuang

**Affiliations:** ^1^ Institute of Medical Research, Northwestern Polytechnical University, Xi’an, Shaanxi, China; ^2^ Department of Immunology, Fourth Military Medical University, Xi’an, Shaanxi, China; ^3^ Orthopedic Department of Tangdu Hospital, Fourth Military Medical University, Xi’an, Shaanxi, China

**Keywords:** IL-6, distress, intestinal mucosal barrier, KO mice, apoptosis

## Abstract

**Background:**

Interleukin-6 (IL-6) is essential for maintaining intestinal epithelial homeostasis. Although cold water-immersion restraint (CWIR) stress is commonly used to induce in vivo gastric injury, it also affects intestinal epithelial permeability. Although IL-6 is increased in response to acute physiological and psychological stress, its exact effects on the pathophysiology of the intestinal epithelium in response to acute CWIR stress remain unknown.

**Methods:**

We used IL-6 knockout (KO) mice with acute CWIR modeling to investigate the effect of IL-6 deficiency on intestinal epithelial morphology and pathological damage using histological staining assays under the acute stress. We detected jejunal epithelial apoptosis using TUNEL and standard molecular experiments.

**Results:**

CWIR caused intestinal epithelial damage, which was alleviated by the absence of IL-6, as evidenced by morphological changes and goblet cell and intestinal permeability alteration. IL-6 KO also reduced CWIR-mediated inflammatory levels and improved stress defense. Meanwhile, IL-6 deficiency decreased the intestinal epithelial apoptosis induced by CWIR administration. This IL-6 KO-led effect depended more on mitochondrial AIF signaling rather than the traditional caspase pathway.

**Conclusion:**

As a result, we concluded that acute CWIR-induced severe intestinal damage and jejunal epithelium apoptosis could be alleviated by IL-6 deficiency, implying a protective effect of IL-6 deficiency on the intestines under acute stress. The findings shed new light on treating CWIR-induced intestinal disorders by inhibiting IL-6 signaling.

## Introduction

Stress is the body’s spontaneous response to any changes in the external environment or internal stimulus that may threaten body homeostasis and cause different changes depending on the stimulus’s duration, type, and severity (1). There are two types of stress animal models: physical and psychological stress models (2). Cold water-immersion restraint stress (CWIR) combines cold stress with immobilization and has been studied as a typical physical stress model for causing gastric ulcers and mimicking stress-related mucosal disease (3, 4). Although many CWIR modeling studies focus on gastric injury (5, 6), approximately 95% of peptic ulcers are duodenal (7). Acute CWIR in rats, according to Saunders et al., disrupted colonic epithelial physiology and altered intestinal epithelial permeability in the jejunum (8, 9). The small intestines’ integrity is required for them to resist external stimuli and perform their functions. Villi height and crypt depth are two important indicators that reflect gut digestive and absorption functions and mucosal homeostasis (10). Higher villi in the intestine indicate a more remarkable ability to absorb nutrients, whereas shallower crypts indicate the presence of more mature intestinal cells to enhance secretion (10, 11). Brain-gut axis plays regulatory roles in the stress-induced intestinal epithelium permeability and mucosal dyshomeostasis (12). Although acute stress frequently causes gastrointestinal (GI) dysfunction, few studies have reported CWIR-induced intestinal damage and manifestations. We previously uncovered that acute CWIR resulted in damaged intestinal mucosa and decreased gut microbiota diversity (13), implying that stress-induced intestinal disorders exist.

Interleukin-6 (IL-6) is a pleiotropic cytokine that regulates various biological processes in the body (14). IL-6 can be secreted by multiple non-immune cells, including epithelial cells and adipocytes (15, 16). It causes systemic and local responses to various stimuli, acting as a paracrine and an endocrine factor (17). Notably, IL-6-dominant activities have dual effects on inflammatory progression, including classic anti-inflammatory signaling *via* membrane-bound IL-6 receptor (IL-6R) and proinflammatory trans-signaling *via* soluble IL-6R (18). Studies demonstrated that IL-6 levels in the blood increased dramatically under both acute physiological and psychological stress conditions; however, there are some issues still worthy of discussion (17, 19). Qing et al. revealed that acute stress induced IL-6 is produced from brown adipose tissues to promote hepatic gluconeogenesisa in mice, but as a stress hormone, its effects on various stimuli types and targeted tissues remain open questions (19, 20).

Moreover, IL-6 is also crucial to maintain intestinal homeostasis. Studies have found that IL-6 suppression causes decreased proliferation of intestinal epithelial cells and poor wound repair (21). Tight junction structures of intestinal epithelial cells were also regulated by IL-6 through channel forming protein claudin-2 (22). Studies informed that small intestinal crypt homeostasis is regulated by autocrine IL-6 from the intestinal epithelium (23). These studies suggest that IL-6 plays an important role in regulating intestinal epithelial development and homeostasis, but relatively little work has focused on the roles of IL-6 in intestinal epithelial pathophysiology under acute stress, particularly CWIR. Here, we mainly investigate the effects of IL-6 knockout (KO) and CWIR and their combination on the intestinal pathological alteration and epithelial apoptosis, especially in the jejunum. Since acute CWIR could occur in some special working populations (such as military, lifeguards, etc.) or extreme distress situations (such as floods, snowstorms, etc.) in reality, this work may provide more theoretical basis for the treatment of CWIR-related intestinal disorders *via* IL-6 signaling.

## Materials and methods

### Animal administration

According to the Guide for the Care and Use of Laboratory Animals, animal experiments were performed. The whole study was reviewed and approved by the Ethical Committee on Animal Experimentation of Fourth Military Medical University (approval number: 20200507). Male wild-type C57BL/6 mice (WT) and IL-6 gene knockout mice (IL-6 KO) with a C57BL/6 background (#C57BL/6J-IL6^tmKopf^) at the age of 8-12 weeks (weighing around 24.5 ± 1g) were acquired from the Animal Center of the Fourth Military Medical University. Standard PCR of murine tail tip tissues was performed for genotype identification of IL-6 KO mice. Western blot of small intestinal tissues was used to check IL-6 protein levels in the knockout mice. All animals were housed in a 12 h day-night cycle with free access to water and food at 22 ± 1°C and 60 ± 10% humidity. The mice in corresponding groups were administrated with acute CWIR for 1 h using the same protocol described in our previous study (13). Briefly, mice were restrained in polyvinyl chloride tubes and vertically immersed in cold water with a temperature around 10°C, at the level of sternum xiphoid. Meanwhile, the mice in the control group were placed in the cages under the same external environment but with no access to water and food. Mice were sacrificed following CWIR, and the blood sample and intestinal epithelial tissues were collected for further experiments. Serum levels of IL-6 in mice after modeling were detected using the mouse IL-6 ELISA kit (Hengyuan Biological, Shanghai, China) according to the manufacturer's instructions.

### Intestinal morphology and histological analysis

The small intestines were extracted and divided into three parts (P-proximal, M-middle, and D-distal). The proximal 0.5 cm segment of the M part was adopted as jejunal tissues. Duodenum was taken in the proximal 0.2 cm segment of the P part, and ileum was the distal 0.5 cm segment of the D part of small intestine. After fixation and dehydration, paraffin-embedded intestinal tissue slices (5 µm) were stained with hematoxylin-eosin (HE, #C0105M, Beyotime, Shanghai, China) and Periodic Acid-Schiff (PAS, #C0142M, Beyotime) dyes. The slices were observed and captured using an EVOS M7000 imaging system (Thermo Fisher Scientific, Waltham, MA, USA). For HE staining, villi height and crypt depth were measured in 10 randomly chosen villi and crypts in each slide from the duodenum, jejunum, and ileum tissue samples. Histopathological scores of each slice of these tissues were blindly evaluated by two technicians based on the Chiu’s scoring criteria described previously (24). Briefly, the criteria are as follows: 0 = normal intestinal mucosal villi, 1 = development of Gruenhagen’s space at the tip of villi with congested capillary, 2 = enlarged space between intestinal epithelial layer and lamina propria, 3 = bare lamina propria and epithelial shedding, 4 = bare lamina propria with villi shedding, dilated and congested capillary, and inflammatory infiltration, 5 = haemorrhage and ulceration of intestinal mucosa and disintegration of lamina propria. Moreover, the average amounts of goblet cells in six random villus-crypt units in each slice from PAS staining were calculated. Each slice was quantified using ImageScope software (Leica Biosystems, Nussloch, Germany) and Olympus OlyVIA (Tokyo, Japan).

### Transmission Electron Microscopy (TEM)

To observe the intestinal epithelial tight junctions, the TEM assay was conducted in jejunal tissues. Briefly, jejunal tissues were pre-fixed in the Fixative for TEM (#G1102, Servicebio, Wuhan, China) and post-fixed with 1% osmium tetraoxide, followed by dehydration with ethanol and embedding in epoxy resin. Tissues were then sliced into 60-80 nm and fished out on the cuprum grids (150 meshes with formvar film). After staining with uranium acetate and lead citrate, the ultrastructure of the intestinal tissues was observed under a transmission electron microscope (Hitachi HT7800, Tokyo, Japan).

### Measurement of gut mucosal permeability

Fluorescein isothiocyanate (FITC)-dextran was employed to determine the intestinal mucosal permeability as previously described (25, 26). Briefly, a total of 12.5 mg FITC-dextran (MW 4000, #R-FD-001, Ruixi Biological Technology, Xi’an, China) was oral-administrated in each mouse after CWIR. Retro-orbital blood collection was performed two hours later, and mice were anesthetized by isoflurane inhalation to avoid additional stimulation to the mice during blood drawing. Serum FITC-dextran concentration was detected using a multimode plate reader (Infinite M Plex, Tecan, Männedorf, Switzerland).

### Real-Time quantitative PCR (RT-qPCR)

TRIGene reagent (GenStar, Beijing, China) was used to extract total RNAs from the jejunal epithelium in each group, which were further reverse-transcribed into cDNAs through Hifair II 1st Strand cDNA Synthesis Kit (Yeasen Biotechnology, Shanghai, China). SYBR qPCR Master Mix (GenStar) was adopted to perform subsequent qPCR with specific murine primers (Supplementary Table 1). β-actin was used as an internal reference, and the 2^−ΔΔCt^ method was adopted to calculate the relative mRNA expression of each gene.

### Immunofluorescence staining and Terminal deoxynucleotidyl Transferase (TdT) dUTP Nick-End Labeling (TUNEL) assay

Paraffin-embedded small intestinal tissue (jejunum) slices were adopted to perform immunofluorescence staining of apoptosis-inducing factor (AIF). Briefly, after fixation and permeabilization, the slices were incubated with primary anti-AIF (#17984-1-AP, Proteintech, Wuhan, China) at 4°C overnight in a wet box, followed by incubation with Cy3–conjugated secondary antibodies for 1 h at room temperature in the dark. TUNEL staining of intestinal tissues was performed to detect cell apoptosis and DNA damage through an *In Situ* Cell Death Detection Kit (Roche Diagnostics, Indianapolis, IN, USA), following the manufacturer’s instructions. Similarly, after fixation and permeabilization, a TUNEL reaction mixture containing enzyme solution and labeling solution was prepared to incubate the slice for 1 h at 37°C in the dark. 4′,6-diamidino-2-phenylindole dihydrochloride (DAPI, #AR1177, BOSTER, Wuhan, China) was used for nuclei staining. Following mounting, the slices were observed and photographed under a fluorescent microscope (EVOS M7000 imaging system, Thermo Fisher Scientific). The average number of TUNEL positive cells per villi in four random field in each slice was counted and analyzed.

### Western blot

Total proteins from jejunal epithelium were extracted through RIPA lysis buffer (#AR0108, BOSTER). The mitochondrial proteins were extracted using the Tissue Mitochondria Isolation Kit (Beyotime). A BCA Protein Assay Kit (Beyotime) quantified protein concentration following the manufacturer’s protocols. Certain amounts of proteins (~30 μg) were loaded onto each lane of SDS-PAGE gel to undergo electrophoresis, then transferred onto PVDF membranes (Millipore, Bedford, MA, USA). After blocking and washing, the membranes were incubated with specific primary antibodies (below) at 4°C overnight. Membrane incubation of corresponding HRP-conjugated secondary antibodies was conducted at room temperature for 1 h. Subsequently, ECL reagents (#E170-01, GenStar) were prepared and added to the membranes for chemiluminescence, which were imaged and captured using a ChemiDoc Touch imaging system (BIO-RAD, Hercules, CA, USA). Primary antibodies used in this study were: Occludin (#AF7644, Beyotime), Claudin 1 (#28674-1-AP, Proteintech), Claudin 2 (#AF0128, Affinity, Changzhou, China), IL-6 (#500286, ZEN BIO, Chengdu, China), Caspase-3 (#19677-1-AP, Proteintech), Caspase-9 (#10380-1-AP, Proteintech), AIF (#AA306, Beyotime), Calpain-1 (#381868, ZEN BIO), Cox-IV (#200147, ZEN BIO), and β-actin (#T0022, Affinity) (internal reference for total protein).

### Statistical analysis

Results are exhibited as mean ± standard error of the mean (SEM). Each experiment contained at least three repeats, and the animal number used in each experiment was stated in the corresponding figure legends. Data were analyzed using the GraphPad Prism 9.0 (GraphPad Software, La Jolla, CA, USA). Means were compared using two-way ANOVA with the Tukey *post-hoc* test, to analyze the effects from genotype, stress condition, and their interaction on the intestinal changes. The *F*_(DFn, DFd)_ and *p* values were indicated in the corresponding context. The scoring-related analysis was conducted by Kruskal-Wallis test with Dunn’s multiple comparisons. The statistical significance was set at *p* < 0.05.

## Results

### Morphological observation of intestinal tissues in IL-6 KO mice with CWIR

**Figure 1:**
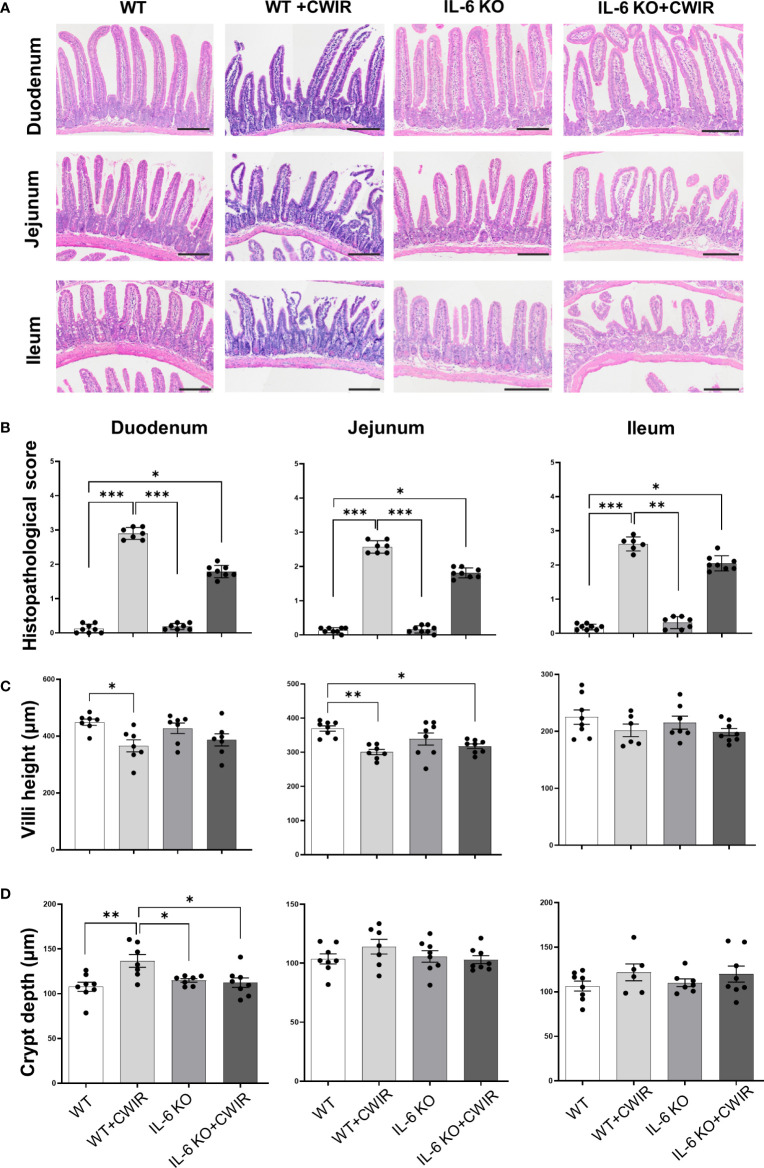
Morphological observation of intestinal tissues in IL-6 KO mice administrated with CWIR. **(A),** Representative images of HE staining of the duodenum, jejunum, and ileum tissues among groups. The scale bar means 200 μm. **(B),** Histopathological scoring analysis of the groups’ HE staining images of the duodenum, jejunum, and ileum tissues. Kruskal-Wallis test with Dunn’s multiple comparisons was used for data analysis. **(C, D)** Measured villi height and crypt depth in the small intestinal tissues in the WT and IL-6 mice with CWIR. Data analysis was performed by two-way ANOVA with the Tukey *post-hoc* test. N = 6-8, *p < 0.05, **p < 0.01, ***p < 0.001.

Since IL-6 was reported to be the dominant cytokine induced by acute stress in mice (19), serum levels of IL-6 before and 0.5-12 h after CWIR in WT mice were measured firstly (Supplementary **Figure 1A**). Results showed that the IL-6 levels in the CWIR group were significantly higher than that in WT, and reached the highest level at 2 h after modeling, then decreased gradually. However, there was no significant difference of them between 0 h and 0.5-4 h after CWIR modeling. Additionally, time-course of jejunal histology in WT mice after CWIR (1-4 h) also showed that although the jejunal villi structure was damaged after stress, there was no obvious difference in various time periods (**Supplementary Figure 1B**). To further investigate the exact roles of IL-6 played after acute CWIR stress, we adopted IL-6 KO mice in subsequent studies. IL-6 KO mice were identified through genotyping PCR and western blot, both of which showed efficient knockout effects of IL-6 (**Supplementary Figure 2**). After administration with acute CWIR, HE staining of duodenum, jejunum, and ileum tissues in each group was performed. Results showed that CWIR led to incomplete epithelium structures with fractured villi and development of Gruenhagen’s space at the villi tips. Exposed lamina propria and some submucosal edema were also observed. IL-6 KO restored CWIR-induced intestinal damages to relatively normal levels, and further CWIR aggravated them (**Figure 1A**). The corresponding histopathological scoring system of small intestinal tissues quantified the intestinal damage and average scores in the duodenum, jejunum, and ileum tissues and indicated that CWIR-caused intestinal injury in small intestines could be relieved by IL-6 KO (**Figure 1B**). To evaluate the main effects on the intestinal morphology, we performed two-way ANOVA for villi height and crypt depth in the intestinal tissues of all four groups. With regard to villi height, there was a significant main effect of CWIR in the duodenum (*F*_(1, 24)_ = 11.18, *p* < 0.01) and jejunum (*F*_(1, 27)_ = 16.32, *p* < 0.001). A significantly reduced villi height was observed in both duodenum and jejunum of WT mice after CWIR (**Figure 1C**). Besides, compared to WT group, IL-6 KO mice with CWIR administration showed significantly reduced villi height in jejunum (**Figure 1C**), resulting in a significant interaction effect between genotype and the stress (*F*_(1, 27)_ = 4.65, *p* < 0.05). Neither IL-6 KO nor CWIR affected ileal villi height (**Figure 1C**). In addition, CWIR in WT mice significantly elevated crypt depth of duodenum, while IL-6 KO reduced the crypt depth to a relatively normal level with or without CWIR (**Figure 1D**). We found a significant main effect of CWIR (*F*_(1, 26)_ = 6.33, *p* < 0.05) and a clear interaction between CWIR and IL-6 KO (*F*_(1, 26)_ = 8.93, *p* < 0.01) in duodenal crypt depth. Other small intestinal tissues showed comparable crypt depth among groups (**Figure 1D**). From above results, CWIR, rather than IL-6 KO, causes severer intestinal pathological damages.

### CWIR-caused increase of intestinal permeability is relieved by IL-6 KO in mice

**Figure 2:**
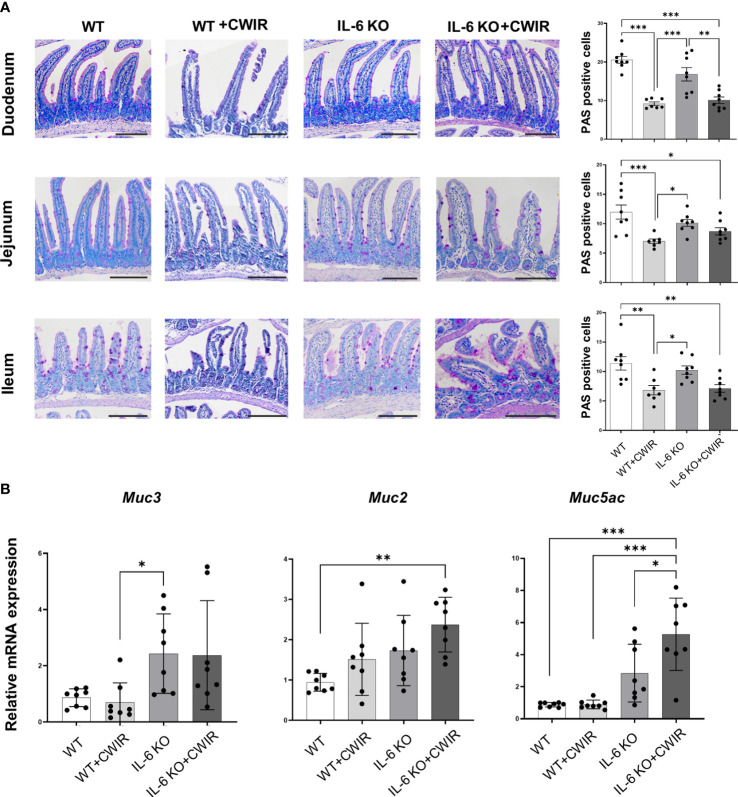
IL-6 KO alleviated the CWIR-induced decrease of goblet cells. **(A),** Goblet cells were stained by PAS staining. Representative images and the quantification of PAS positive cells were exhibited. The scale bar means 200 μm. N=7-8. **(B),** Relative mRNA levels of *Muc3*, *Muc2*, and *Muc5ac* in the jejunum among groups. N=8-10. Data analysis was performed by two-way ANOVA with the Tukey *post-hoc* test. *p < 0.05, **p < 0.01, ***p < 0.001.

**Figure 3:**
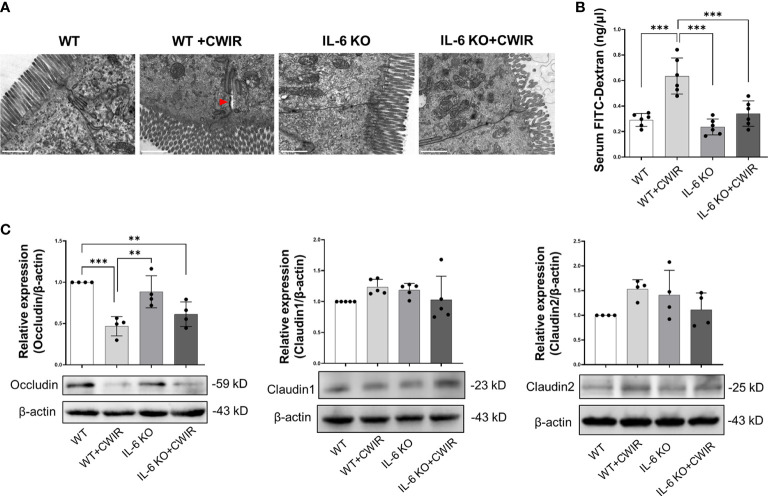
CWIR damaged intestinal epithelial tight junctions, which was improved by IL-6 KO. **(A),** Representative TEM images of jujunal tissues. Red arrow indicates the wider interval between epithelial cells. The scale bar means 10 μm. N=3. **(B),** Serum FITC-dextran concentration was evaluated in each group. N=6. **(C),** Relative protein expressions of occludin, claudin 1, and claudin 2 in the jejunum tissues among groups were checked and quantified. N=4-5, Data analysis was performed by two-way ANOVA with the Tukey *post-hoc* test. **p < 0.01, ***p < 0.001.

We then performed PAS staining to observe the changes of goblet cells in the duodenum, jejunum, and ileum tissues of WT and IL-6 KO mice under CWIR (**Figure 2A**). Quantification of PAS positive cells showed that compared to WT mice, CWIR led to reduced goblet cell amounts in all small intestinal tissues. IL-6 KO mice showed similar reduction after CWIR. Significant main effects of CWIR were found in all intestinal tissues (Duodenum: (*F*_(1, 27)_ = 64.89, *p* < 0.001); Jejunum (*F*_(1, 27)_ = 16.74, *p* < 0.001); Ileum (*F*_(1, 27)_ = 20.59, *p* < 0.001)), and clear interactions were shown in the duodenum (*F*_(1, 27)_ = 4.31, *p* < 0.05) and jejunum (*F*_(1, 27)_ = 5.16, *p* < 0.05). However, IL-6 deficiency alone did not affect goblet cell amounts in the small intestinal tissues compared to WT. The findings indicated that IL-6 KO may alleviate CWIR-induced goblet cell reduction (**Figure 2A**), which was similar to the alterations of intestinal pathological damages. We next detected the relative mRNA expression of mucins in jejunum among groups. **Figure 2B** showed that compared to WT with CWIR, IL-6 KO significantly increased Muc3 (a trans-membrane mucin) expression; however, no significant change was observed when IL-6 KO with CWIR. A main effect of genotype was shown in Muc3 expression (*F*_(1, 28)_ = 13.3, *p* < 0.01). Meanwhile, gel-forming mucins Muc2 and Muc5ac in IL-6 KO mice with CWIR were markedly elevated compared to WT. Muc5ac in IL-6 KO+CWIR groups were also significantly higher than that in WT+CWIR and IL-6 KO groups (**Figure 2B**). There were significant main effects of CWIR (*F*_(1, 28)_ = 5.68, *p* < 0.05) and genotype (*F*_(1, 28)_ = 10.55, *p* < 0.01) in Muc2 expression, as well as CWIR (*F*_(1, 28)_ = 5.67, *p* < 0.05), genotype (*F*_(1, 28)_ = 38.24, *p* < 0.001), and an obvious interaction (*F*_(1, 28)_ = 5.47, *p* < 0.05) in Muc5ac expression. It seems that IL-6 KO played major roles in modulating mucin expression levels of jejunum. Moreover, we observed the ultrastructure of the intestinal epithelial tight junctions through TEM, which revealed that CWIR damaged the tight junctions between intestinal epithelial cells in WT mice, which was obviously improved when IL-6 deficiency (**Figure 3A**). We also detected the gut leakage by FITC-dextran administration (**Figure 3B**). The serum concentration of FITC-dextran was significantly increased in WT mice with CWIR (significant main effect of CWIR, *F*_(1, 20)_ = 33.26, *p* < 0.001), and compared with that, IL-6 KO mice showed obviously reduced FITC-dextran with or without CWIR (significant main effect of genotype, *F*_(1, 20)_ = 20.19, *p* < 0.001; significant interaction effect, *F*_(1, 20)_ = 9.50, *p* < 0.01). Moreover, CWIR also led to a significant decrease in occludin protein expression, a staple of tight junction, in the jejunum, which was relieved by IL-6 KO (**Figure 3C**, significant CWIR effect, *F*_(1, 12)_ = 35.03, *p* < 0.001). However, protein expression levels of claudin 1 and claudin 2 showed no big difference in the jejunum among groups (**Figure 3C**), but significant interaction effects were observed in the claudin 1 (*F*_(1, 16)_ = 4.54, *p* < 0.05) and claudin 2 (*F*_(1, 12)_ = 6.85, *p* < 0.05) expressions. Taken together, the results suggest that IL-6 KO alleviates the CWIR-induced decrease of goblet cell production and elevated gut leakage in small intestines.

### IL-6 KO alleviates CWIR-mediated inflammation and enhances defense under CWIR

**Figure 4:**
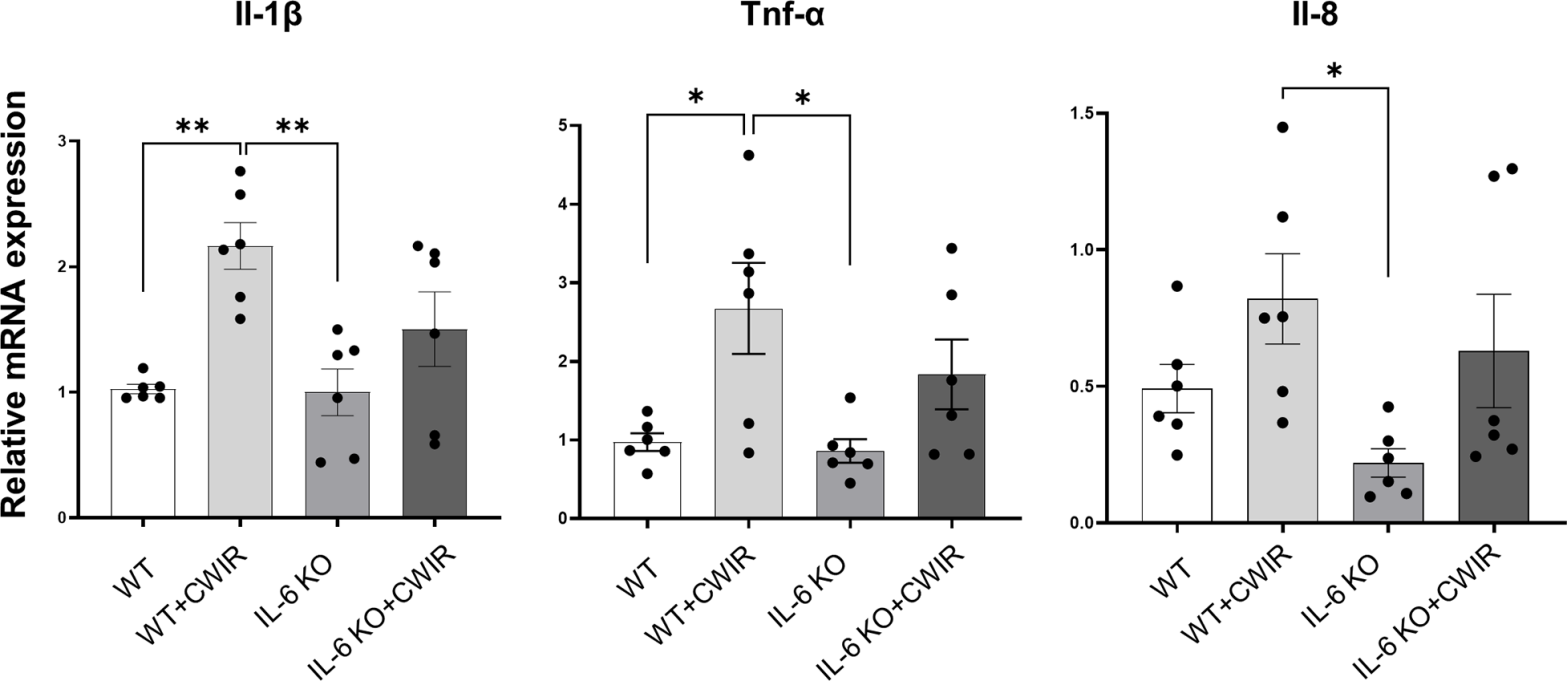
IL-6 KO alleviated CWIR-mediated inflammatory cytokine expression in the jejunum. Relative mRNA levels of proinflammatory cytokines, including *Il-1β*, *Tnf-α*, and *Il-8*, were evaluated. Data analysis was performed by two-way ANOVA with the Tukey post-hoc test. N=6, *P < 0.05, **P < 0.01.

**Figure 5:**
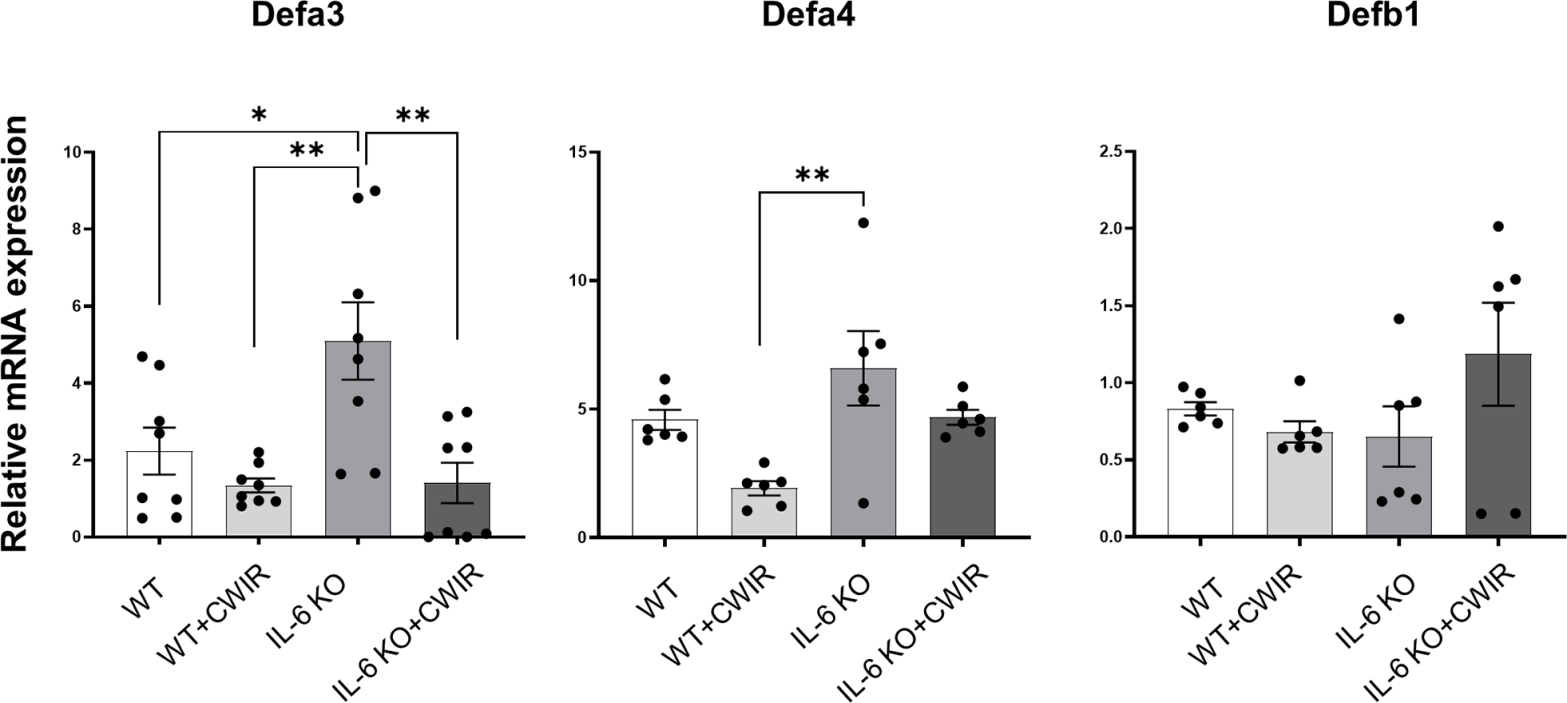
IL-6 KO alleviated CWIR-mediated enhanced defensins expression in the jejunum. Relative mRNA levels of *Defa3*, *Defa4*, and *Defb1* among groups. Data analysis was performed by two-way ANOVA with the Tukey post-hoc test. N=6-8, *P < 0.05, **P < 0.01.

To fully understand the effects of IL-6 KO and CWIR on murine intestines, relative mRNA expression levels of several proinflammatory indicators and defensins in jejunum were assessed by RT-qPCR. Results showed that CWIR administration increased the proinflammatory levels, as evidenced by the enhanced expression of *Il-1β, Tnf-α, and Il-8*, whether in WT or IL-6 KO mice, but IL-6 KO did not alter the proinflammatory levels with or without CWIR stress (**Figure 4**). Main effects of CWIR were also found in *Il-1β* (*F*_(1, 20)_ = 17.07, *p* < 0.001), *Tnf-α* (*F*_(1, 20)_ = 12.58, *p* < 0.01), and *Il-8* (*F*_(1, 20)_ = 6.75, *p* < 0.05) expressions. Intriguingly, IL-6 KO showed significantly increased levels of *Defa3* compared to WT, which was further reduced by CWIR. Significant main effects of IL-6 KO (*F*_(1, 28)_ = 5.04, *p* < 0.05), CWIR (*F*_(1, 28)_ = 12.36, *p* < 0.01), and their interaction (*F*_(1, 28)_ = 4.60, *p* < 0.05) were found in *Defa3* expression levels. Although *Defa4* was also the highest in IL-6 KO, there was no significant change compared to WT alone. Nevertheless, both IL-6 KO (*F*_(1, 20)_ = 9.40, *p* < 0.01) and CWIR (*F*_(1, 20)_ = 8.64, *p* < 0.01) effects can affect *Defa4* expression. *Defb1* expression had no noticeable change among the groups (**Figure 5**). The findings suggest that IL-6 KO could alleviate the CWIR-induced elevation of inflammatory cytokines and enhance the defense after CWIR stress.

### IL-6 KO relieves CWIR-induced cell apoptosis in the murine intestine

**Figure 6:**
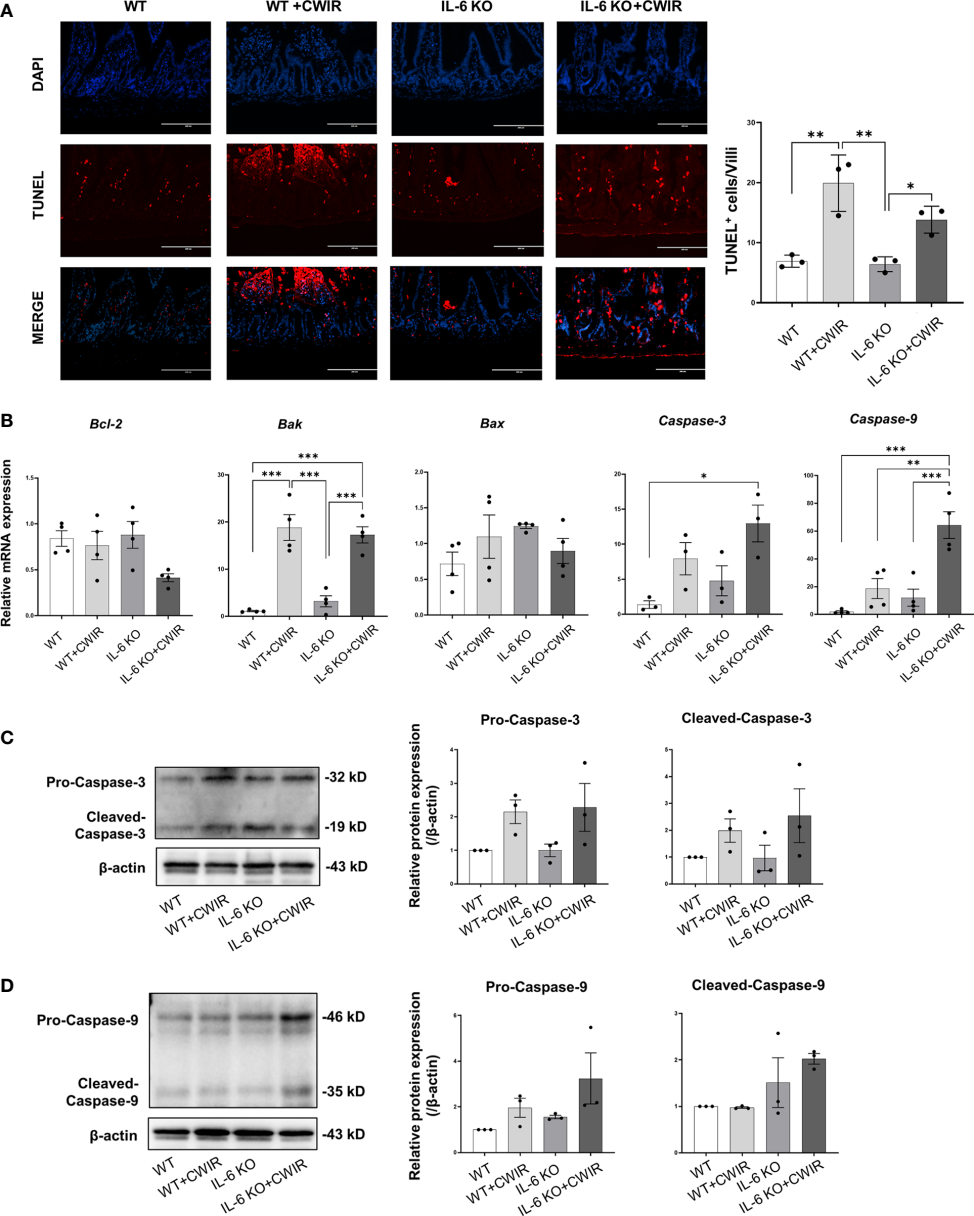
CWIR-caused jejunal epithelium apoptosis was relieved by IL-6 deficiency in mice. **(A),** Representative immunofluorescence micrographs of TUNEL (red) and DAPI (blue) staining in the intestinal epithelium. TUNEL positive cells per villi was quantified and exhibited. The scale bar means 200 μm. N=3. **(B),** The expressions of some apoptosis-related indicators among groups were examined at the transcriptional level. N=3-4. **(C, D),** Western blot results and quantification of pro and cleaved-caspase-3 and caspase-9 among groups. N=3, Data analysis was performed by two-way ANOVA with the Tukey post-hoc test. *P < 0.05, **P < 0.01, ***p < 0.001.

Furthermore, we used the TUNEL assay to measure irreversible cell death in the jejunum with or without CWIR in the presence or absence of the IL-6 gene. Representative images of TUNEL-positive cells in the intestines were shown in **Figure 6A**. Compared with the WT, we observed considerably much TUNEL-positive cells on the top of villi in the CWIR group, while the absence of IL-6 rarely affected cell apoptosis, which was increased again by further CWIR. Interestingly, more apoptotic cells were evenly distributed on the villi in IL-6 KO mice with CWIR (**Figure 6A**). A main effect of CWIR was observed in TUNEL-positive cells on villi (*F*_(1, 8)_ = 42.09, *p* < 0.001). Moreover, we detected the mRNA expression levels of several apoptosis-related molecules within groups. It was shown that although there were slight changes in *Bcl-2* levels in WT mice after CWIR, CWIR in IL-6 KO mice decreased its expression compared to other groups (with a significant main effect of CWIR, *F*_(1, 12)_ = 5.44, *p* < 0.05). The changes in pro-apoptosis *Bak* expression among groups agreed with the results of TUNEL staining and revealed a significant CWIR effect (*F*_(1, 12)_ = 85.35, *p* < 0.001); however, *Bax* expression levels showed no significant difference among groups (**Figure 6B**). Moreover, both *caspase-3* and *caspase-9* levels elevated in IL-6 KO mice with CWIR compared to other groups (**Figure 6B**). CWIR played major roles in modulating *caspase-3* (*F*_(1, 8)_ = 12.69, *p* < 0.01), while *caspase-9* was affected by CWIR (*F*_(1, 12)_ = 26.18, *p* < 0.001), IL-6 KO (*F*_(1, 12)_ = 17.18, *p* < 0.01), and their interaction (*F*_(1, 12)_ = 6.98, *p* < 0.05). We further explored the jejunal protein expression of caspase-3 and 9 in the four groups. Consistent with their mRNA levels, both pro and cleaved-caspase-3/9 showed similar variation trends (**Figure 6C, D**), although there was no statistical significance. A significant CWIR effect was shown in pro-caspase-3 expression (*F*_(1, 12)_ = 10.10, *p* < 0.01), and a IL-6 KO effect in cleaved-caspase-9 expression (*F*_(1, 8)_ = 8.09, *p* < 0.05). It seems that CWIR causes more apoptosis-like cells than IL-6 KO, but IL-6 KO also had crucial impacts on typical apoptosis-related molecule caspase-9.

### IL-6 KO significantly affects mitochondrial AIF signaling to modulate intestinal epithelium apoptosis

**Figure 7:**
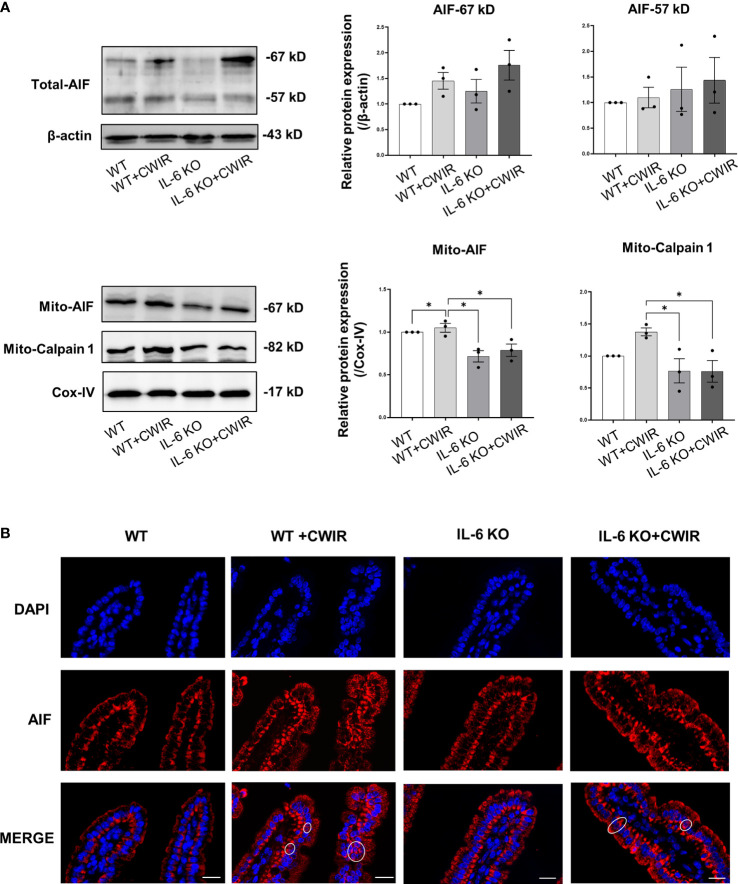
IL-6 KO significantly altered the mitochondria AIF signaling after acute CWIR rather than caspases. **(A),** Western blot images and quantification of total AIF, mitochondrial AIF, and calpain 1 in the intestinal epithelium within the four groups. Data analysis was performed by two-way ANOVA with the Tukey post-hoc test. N=3, *P < 0.05. **(B),** Representative immunofluorescence images of AIF (red) and DAPI (blue) in the jejunal epithelium. Double staining of AIF and DAPI was circled. The scale bar represents 20 μm. N=3.

Since less statistical significance was observed in the classical apoptosis-related caspase3/9 signaling among groups, we further detected AIF signaling that mainly dominate caspase-independent cell apoptosis in groups (27). Results showed that total protein levels of AIF were increased in CWIR groups, and IL-6 KO reduced them, especially for 67 kDa AIF (**Figure 7A**, significant main effect of CWIR in AIF-67 kD, *F*_(1, 8)_ = 5.66, *p* < 0.05). Full-length AIF in the mitochondria could be cleaved to 57 kDa and translocated into the nucleus to regulate cell apoptosis (28). Although the total protein alteration had no statistical significance among groups, mitochondrial AIF in the CWIR group showed the highest level, and IL-6 KO significantly reduced the mitochondrial AIF under both normal and CWIR condition (**Figure 7A**). Meanwhile, the protein levels of calpain 1, an upstream modulator of mito-AIF, showed similar remarkably alterations in mitochondria, in which IL-6 KO significantly diminished the upregulated calpain 1 expression induced by CWIR (**Figure 7A**). Interestingly, the main effects of IL-6 KO were found in mito-AIF (*F*_(1, 8)_ = 24.07, *p* < 0.01) and mito-calpain 1 (*F*_(1, 8)_ = 10.51, *p* < 0.05) expression levels. Further immunofluorescence staining of AIF exhibited apparent nuclear translocation in the CWIR groups, but IL-6 KO showed no obvious translocation (**Figure 7B**), which agreed with the results from TUNEL assay. Hence, mitochondrial AIF signaling might be a dominant strategy for IL-6 to modulate intestinal epithelium apoptosis under CWIR.

## Discussion

Stress induces a variety of physiological and pathological complications in the body, affecting the normal functions of multiple organs, including the GI system (1, 29). Acute CWIR has been widely used in animal models to induce gastric ulcers (3, 5). Meanwhile, evidence suggests that CWIR is a powerful effector of epithelial permeability and barrier function in rat intestines, particularly jejunal tissues (9, 30). As an inflammatory cytokine, IL-6 regulates pathophysiological processes locally and systemically in the body (17). Studies revealed that IL-6 is a crucial mediator to regulate intestinal permeability and growth of intestinal epithelial cells (22, 31, 32). Both CWIR and IL-6 have been studied to alter the permeability of intestinal epithelium; however, the effects of their combination on the intestines of mice have rarely been studied, which drew our attention. This study focused on the effects of IL-6 deficiency on the small intestinal morphology and epithelial apoptosis, especially in jejunum, under acute CWIR conditions.

In this case, CWIR had an apparent effect on villi height in WT mice, but IL-6 KO had no effect. The previous finding showed that villus height of jejunum was elevated in IL-6 systemically administrated mice (33); however, only minor changes in jejunal villi height were observed in IL-6 KO mice in current study. It is worth noting that the crypt depth increased after CWIR in the duodenum of WT mice, and the absence of IL-6 reversed this alteration, resulting in a shallower crypt depth comparable to WT mice with CWIR. Other studies also found comparable crypt depths between IL-6 KO/IL-6 administration and WT (23, 33). Acute CWIR appeared to cause intestinal damage, primarily in the villi, such as villi tip shedding and reduced villi height, while IL-6 deficiency exhibited less effects on the small intestinal histopathological changes.

Goblet cells are important secretory cells in the conjunctival, respiratory, and gastrointestinal epithelium (34). Goblet cells’ primary function is to produce and secrete mucins, which are the primary constituents of the mucus layer (35). The mucus layer, located in the transition zone between the outer and inner epithelium, acts as a solid barrier against the invasion of foreign pathogens (35). CWIR treatment significantly reduced goblet cells, consistent with the disruption of intestinal villus-crypt structures caused by CWIR. We discovered that removing IL-6 alleviated CWIR-induced goblet cell decline and gut leakage, and restored them to levels close to WT, implying that IL-6 deficiency may play a protective role in CWIR-induced intestinal permeability. Previous study found that IL-6 increased intestinal epithelial tight junction permeability by inducing claudin 2 expression in the intestinal epithelial cells Caco-2 (22), but there was no significant change observed of claudin 2 expression in the jejunum among the four groups in this study. However, altered trend of claudin 2 expression showed that it was increased after CWIR which was then reduced by further IL-6 deficiency. Combined with the declined expression of occludin under CWIR, these results conformed the characteristics of claudin 2 molecule that is a “leaky” pore-forming claudin (36). Furthermore, molecular expression changes of mucins in the jejunum were inconsistent. As a typical trans-membrane mucin, Muc3 showed slight decrease after CWIR without statistical significance, but loss of IL-6 significantly reversed this reduction, suggesting that IL-6 loss has predominant function on Muc3 expression in jejunum under CWIR. The difference in the gel-forming mucins, Muc2 and Muc5ac, is that they were significantly elevated in the IL-6 KO mice with CWIR compared to other groups that showed little changes in between. The findings seemed that IL-6 KO played dominant roles in modulating mucin expression levels of jejunum.

IL-1β, TNF-α, and IL-8, on the other hand, have been shown in studies to increase mucin gene expression in the human airway or lung epithelial cells during inflammation (37, 38). Although CWIR significantly reduced the number of goblet cells, the expression levels of *Il-1β*, *Tnf-α*, and *Il-8* were remarkably increased after CWIR. CWIR also showed main effects affecting the expression of these proinflammatory cytokines. Combined with the mucin expression changes, it is hypothesized that CWIR-caused elevation of these proinflammatory cytokines could initiate an inflammatory response and also regulate goblet cell mucin secretion in response to acute stress. However, the addition of IL-6 KO also exerts effects on them. Since the different roles of mucin members in the intestine (39), final expression of them is the results of an interplay of altered goblet cells and inflammatory response. Of course, other factors (like epithelial cells) may be involved in this regulation, which needs further investigation.

Meanwhile, defensins are a class of antimicrobial peptides found in neutrophils, macrophages, and epithelial cells that are primarily classified into α and β- subfamilies (40). Our findings showed that IL-6 KO increased *Defa3* and *Defa4* expression levels in WT mice after CWIR, but there was no significant difference in *Defab1* expression between groups. Because Paneth cells in the small intestine produce a large number of α-defensins (41), the absence of IL-6 may also affect the stress-altered Paneth cell functions. Given that Paneth cells live at the bottom of the crypt (41), although the effects of CWIR on crypt morphology were only observed in the duodenum, the alteration of crypt functions in IL-6 KO under stress warrants further investigation.

Furthermore, jejunal epithelium apoptosis was studied in different groups. Based on the findings, it was determined that IL-6 KO reduced CWIR-induced cell apoptosis in murine intestines. Further molecular experiments revealed that IL-6 knockout had a greater impact on mitochondrial AIF signaling than canonical caspases in regulating cell apoptosis. A previous study found that inhibiting IL-6 reduced intestinal epithelial proliferation (21) and increased intestinal epithelial apoptosis through caspase-3 activation (33). However, IL-6 deficiency showed fewer effects on intestinal epithelial apoptosis and caspase-3 in this study. This may due to other impacts from CWIR. Because CWIR-induced effects on the intestinal epithelium apoptosis were much greater than that induced by IL-6 KO, it is understandable that there was no statistical significance between IL-6 KO and WT mice alone among the four groups. Meanwhile, apart from *Bak* expression, the patterns of gene expression of *Bcl-2*, *Bax*, *caspase-3*, and *caspase-9* do not completely match up with the patterns of TUNEL staining across CWIR and IL-6 KO, although some similar trends were shown. We speculated that the apoptotic cells may also be much affected by other signaling in IL-6 KO mice with CWIR. Because the changes of caspase protein signaling were insignificant, the caspase-independent AIF apoptotic signaling pathway attracted our attention. The mitochondrial AIF signaling increased after CWIR but decreased dramatically in the absence of IL-6. AIF is being studied for its ability to induce cell apoptosis in a caspase-independent manner *via* calpain 1 cleavage and translocate from mitochondria into nucleus (42). Calpain 1 expression changes were significant and consistent with mitochondrial AIF, implying that IL-6 KO affected mitochondrial AIF signaling more than classical caspases to regulate the cell apoptosis after acute CWIR.

Interestingly, we also observed that the apoptotic cells occurred at the top half of the villi of jejunum in WT mice with CWIR, but in IL-6 KO mice with CWIR, they occurred evenly throughout the villus from TUNEL staining. Back to the morphological changes in the two genotype mice after CWIR, WT showed more villi tip shedding but the apical edges of villus remained in IL-6 KO mice, which may be causally related to the different distribution of apoptosis in the jejunal epithelium under different genotype backgrounds. Previous study uncovered that IL-6 administration induced intestinal hyperplasia that was due to prolonged enterocyte lifespan and decreased enterocyte migration rate. The TUNEL staining revealed that IL-6 administration led to more positive cells at the middle-upper part of the jejunal villi compared to the controls. This was associated with the activation of STAT3 in enterocytes along the middle-lower villi (33). Thus, IL-6 KO may alter the enterocyte migration rate in the jejunum, leading to various distribution of apoptosis. Meanwhile, in order to maintain the intestinal homeostasis, IL-6 KO mice occurred some physiologically compensatory alterations, such as reduced gut microbiota diversity and increased intraepithelial lymphocytes (43). This may enhance the anti-stress capability of intestinal epithelial cells in IL-6 KO mice, so less apoptosis was shown on the jejunal villi of IL-6 KO mice under CWIR. Further exploration would be considered to verify these effects.

Finally, IL-6 KO alleviated acute CWIR-induced intestinal damage and epithelium apoptosis, implying IL-6 deficiency protects the intestines under acute stress. Because acute CWIR can occur in unusual environments and workgroups, such as floods, soldiers, and lifeguards, the findings offer a novel strategy for the treatment of CWIR-related intestinal disorders by inhibiting IL-6 signaling.

## Data availability statement

The raw data supporting the conclusions of this article will be made available by the authors, without undue reservation.

## Ethics statement

The animal study was reviewed and approved by Ethical Committee on Animal Experimentation of Fourth Military Medical University.

## Author contributions

YZ and CD: manuscript drafting, study design, and statistical analysis. SW, JM, YL, and WL completed all laboratory work. TW and LY helped plan and coordinate the study. KC: mice genotyping and preparation. RZ: study design and administration support. All authors contributed to the article and approved the submitted version.

## Funding

This work was supported by the Natural Science Basic Research Program of Shaanxi (Program No. 2021JM-081) and the funds provided by Fourth Military Medical University (Grant Nos. 2018HKPY02 and 2020rcfczr).

## Conflict of interest

The authors declare that the research was conducted in the absence of any commercial or financial relationships that could be construed as a potential conflict of interest.

## Publisher’s note

All claims expressed in this article are solely those of the authors and do not necessarily represent those of their affiliated organizations, or those of the publisher, the editors and the reviewers. Any product that may be evaluated in this article, or claim that may be made by its manufacturer, is not guaranteed or endorsed by the publisher.
