# Supplementary material for: Knockout of IL-6 mitigates cold water-immersion restraint stress-induced intestinal epithelial injury and apoptosis
Source: Front Immunol. 2022 Nov 25;13:936689. doi: 10.3389/fimmu.2022.936689 (PMC9732082; doi:10.3389/fimmu.2022.936689)
Supplement: Supplementary file 1 [file Presentation_1.pdf]

Supplementary Material

Supplementary Figures

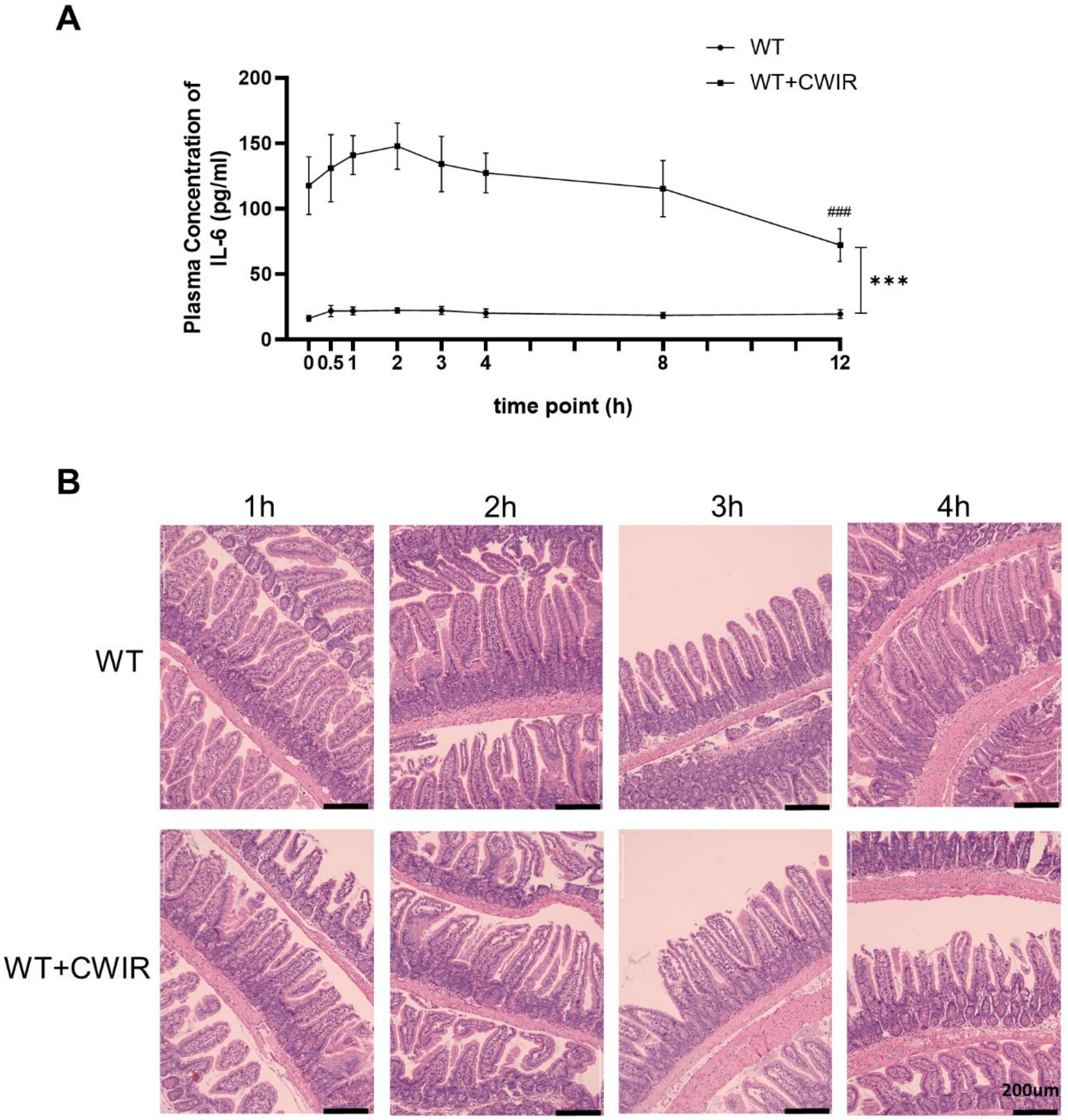

Supplementary Figure 1. (A), Serum levels of IL-6 levels before and 0.5-12 h after CWIR in mice by ELISA. N=3 in 0 h and N=6 in other time point. $^{\#\#}P < 0.01$ vs. the WT+CWIR in 0 h; $^{***}p < 0.001$. **(B)**, HE staining of jejunal histology in WT mice after CWIR (1-4 h). The scale bar means 200 μm. N=6.

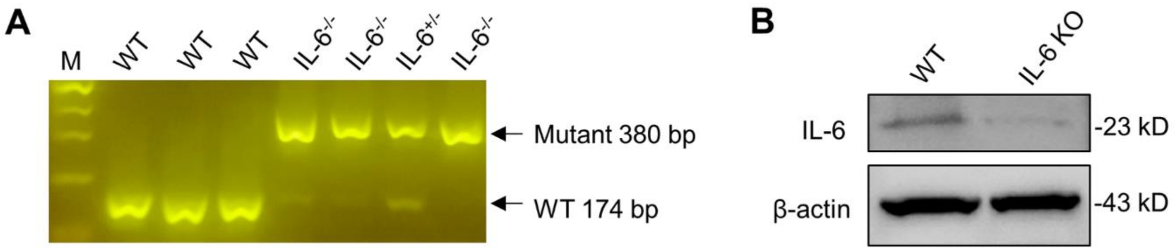

Supplementary Figure 2. Animal identification of IL-6 KO mice was conducted through standard PCR (A) and western blot (B).

Supplementary Table 1. PCR primer sequences.

Primers	5′–3′	Sequences
Il-1β	Forward	TGGACCTTCCAGGATGAGGACA
	Reverse	GTTCATCTCGGAGCCTGTAGTG
Tnf-α	Forward	CAGGCGGTGCCTATGTCTC
	Reverse	CGATCACCCCGAAGTTCAGTAG
Il-8	Forward	GGTGATATTCGAGACCATTTACTG
	Reverse	GCCAACAGTAGCCTTCACCCAT
Defa3	Forward	CTAAAACTGAGGAGCAGCCAGG
	Reverse	GCCTCTTTTTCTACAATAGCATACC
Defa4	Forward	CTAATACTGAGGAGCAGCCAGG
	Reverse	GCAGTGTCCTTTTCTACAATAGCA
Defb1	Forward	AGGTGTTGGCATTCTCACAA
	Reverse	GCTTATCTGGTTTACAGGTTCCC
Bcl-2	Forward	CCTGTGGATGACTGAGTACCTG
	Reverse	AGCCAGGAGAAATCAAACAGAGG
Bak	Forward	GGAATGCCTACGAACTCTTCACC
	Reverse	CAAACCACGCTGGTAGACGTAC
Bax	Forward	AGGATGCGTCCACCAAGAAGCT
	Reverse	TCCGTGTCCACGTCAGCAATCA
Caspase-3	Forward	GGAGTCTGACTGGAAAGCCGAA

	Reverse	CTTCTGGCAAGCCATCTCCTCA
Caspase-9	Forward	GCTGTGTCAAGTTTGCCTACCC
	Reverse	CCAGAATGCCATCCAAGGTCTC
Muc2	Forward	GAGTAGAAGTCCCGAAGGA
	Reverse	ACAATGTTGATGCCAGACTCG
Muc3	Forward	AAAGATTACCTCCCATCTCC
	Reverse	TAAAACTAAGCATGCCCTTG
Muc5ac	Forward	CCACTTTCTCCTTCTCCACACC
	Reverse	GGTTGTCGATGCAGCCTTGCTT
β-actin	Forward	CATGTACGTTGCTATCCAGGC
	Reverse	CTCCTTAATGTCACGCACGAT
